# Tips for a Successful Global Health Sabbatical

**DOI:** 10.5334/aogh.724

**Published:** 2019-05-09

**Authors:** Jason T. Blackard

**Affiliations:** 1 Division of Digestive Diseases, University of Cincinnati College of Medicine, US

## Abstract

There is a high demand for global health experiences among medical students. However, creating and engaging successful global health experiences for students is not without significant challenges for faculty. Lack of broad global health knowledge, first hand experiences in a variety of global health settings, and limited faculty engagement in global health activities at their home institutions may limit overall effectiveness. Thus, sabbaticals may provide unique opportunities for faculty reinvigoration and development of new teaching and/or research activities in global health. This viewpoint stems from my sabbatical experiences in South Africa and Botswana during the 2016–17 academic year and includes important tips for ensuring a successful global health sabbatical.

*Sabbatical* is frequently defined as a paid leave for professional and/or personal development. The benefits of sabbaticals are many and include reflection and renewal, re-evaluation of career goals, intellectual stimulation, and enhanced academic productivity. This can include manuscript and grant writing, networking with colleagues, and overall professional growth. While senior faculty – particularly in medical specialties – rarely take sabbaticals [1,2,3], these formal or informal experiences provide a unique opportunity for faculty re-invigoration and development of new teaching and/or research activities in global health. Global health sabbaticals may facilitate new collaborations in teaching, clinical service, and research, and should benefit both the home and the host institutions as much as possible. Another benefit of global health sabbaticals is enabling faculty to address the prolific student demand in this area. For example, medical student interest in global health opportunities has risen dramatically in recent years [4,5,6]. Medical schools have responded by developing courses domestically and abroad, expanding international partnerships, and engaging in global health research activities. A survey of US medical schools reported that 87% offered international clinical electives, 57% offered clinical research opportunities, and 11% offered a formal global health track for learners [7]. Of US medical schools, 68% have active global or international health student interest groups [6]. While global health has become an integral part of the competitive landscape for most medical schools, 35–40% of medical students describe the instruction they received in global health issues as “inadequate” [8]. There is also a high demand for global health experiences among medical students enrolled in non-US institutions [9], although considerable work remains to provide robust global health opportunities for students to meet the rapidly expanding interest.

The growing learner interest in global health represents an opportunity for educators to teach principles of public health and preventive medicine that are relevant to nearly every patient encounter and in all populations. These experiences are often transformative for students and lead many to choose primary care specialties, engage in community service, and/or work with vulnerable or marginalized populations [5]. Moreover, international opportunities in global health education, research, or clinical work can serve as important recruitment tools to attract the most competitive learners at all levels [5]. However, creating successful global health experiences for students is not without significant challenges for the faculty that facilitate such activities. Lack of broad global health knowledge, first hand experiences in a variety of global health settings, and limited faculty engagement in global health activities at their home institutions may limit overall effectiveness. Faculty experience is frequently inadequate owing to the simple fact that many were trained before structured global health programs existed. Similarly, recent efforts to develop global health competencies for trainees may not adequately address the same needs for faculty and often lack direct input from the host institutions or resource-limited countries [10,11,12,13,14]. Thus, sabbaticals may provide unique opportunities for faculty re-invigoration and development of new teaching and/or research activities in global health. One could also argue that in the interest of cultivating strong north-south collaborations, an imperative exists for faculty engaged in global health work to spend time in resource-limited settings. Surprisingly, literature on sabbaticals is quite sparse and even fewer focus on international or biomedical-focused sabbaticals. For instance, a 2018 search of PubMed yielded only four hits for the search term *global health sabbatical*. In this discussion, I provide the essential guideposts for initiation and successful completion of a global health sabbatical.

I am a PhD-trained virologist with a long-standing interest in viral co-infections. My research laboratory regularly conducts population-based studies of viral infections including studies of pathogen diversity in different at-risk populations, drug resistance, and emerging viral pathogens or new variants of established viruses. I began my academic career in 2005 when I was hired as a Research Assistant Professor at the University of Cincinnati College of Medicine. Several years later, after expanding my research program, mentoring a number of trainees, and being promoted, I sought opportunities to re-invent my research program to focus specifically on clinically relevant research in resource-limited countries and/or conducted with vulnerable populations. Additionally, I wanted to expand my research portfolio to include emerging infections that were not studied regularly at my own institution. I also sought to update my classroom lecture and experiential learning materials to include the most up-to-date, real world examples possible. Finally, I needed time for some adventure and self-reflection to stimulate further personal and professional development.

For several years, I have been working with the Department of Virology at Sefako Makgatho Health Sciences University (formerly the University of Limpopo at MEDUNSA) in Pretoria, South Africa. This department includes a number of clinical and translational researchers focused on infections such as HIV, hepatitis B virus (HBV), hepatitis C virus (HCV), and rotavirus that are common in this part of the world. In collaboration with the previous chair of the department in South Africa, I had submitted and been awarded an NIH grant to study occult HBV in HIV co-infected individuals in South Africa. These studies in South Africa and expertise in viral hepatitis were also of interest to the Botswana-Harvard AIDS Partnership in Gaborone, Botswana. This collaboration was initiated in the late 1990s by my PhD advisor (Dr. Max Essex) and others at Harvard, so I was aware of their research infrastructure and the growing attention being paid to common co-infections such as HBV and HCV in persons with HIV. Thus, it was quite natural for me to conduct my global health sabbatical at these two institutions. This experience would also enable completion of several outstanding manuscripts, in-person training aimed at capacity building within these institutions, and focused time with collaborators to discuss new grant opportunities and additional research training needs.

The tips described below are designed for faculty interested in arranging their own global health sabbatical and stem from my sabbatical experiences in South Africa and Botswana during the 2016–17 academic year. During this time, I spent approximately seven months in southern Africa on four separate trips ranging in length from three weeks to three months.

1. **Define the sabbatical objectives clearly while keeping them flexible.** First, endeavor to develop sabbatical goals in partnership with your host partners. Recognize that your personal and professional objectives may not overlap perfectly with the expectations of your host institution; however, aligning them as much as possible can prevent misunderstandings and engender a more cooperative and collaborative environment. Defining these objectives starts with a thorough review of your own institution’s policies related to sabbatical leave. As an example, the University of Cincinnati’s academic leave application requires the following elements:
  - Briefly describe what project(s) you will undertake on your proposed leave;
  - List the products/accomplishments you aim to achieve through this leave if it is approved;
  - Briefly but specifically describe how your proposed leave will advance your professional development;
  - Briefly but specifically describe how your proposed leave will advance the university’s academic interests in the production and dissemination of knowledge;
  - Briefly but specifically describe how your duties (teaching, mentoring of graduate students, service, etc.) will be covered during your leave.
  Sabbatical applicants must submit a current CV, letters of invitation from host institutions if relevant, and any supplemental information regarding the proposed leave project that is necessary to evaluate the proposal. While my objectives changed somewhat over time, it was important to keep in mind the needs of my host institutions as much as possible. They were specifically interested in completing manuscripts that were already in progress, writing new grant applications for domestic and international funding agencies, mentoring junior investigators, and strengthening their research capacity through training seminars and workshops. Personal goals should not be minimized and can be integrated with professional goals. Several of my personal objectives were making additional friends, adapting to being away from friends and family for an extended period, getting to know my work colleagues better, and visiting particular places that I had not visited previously. Setting clear objectives further facilitates sabbatical preparation and planning.
2. **Prepare well in advance.** My university preparation started approximately one year in advance of the actual sabbatical start date. This included setting reasonable objectives and deliverables, discussing initial objectives with a variety of individuals within and beyond my own institution, completing the three-page online sabbatical application, requesting letters of support of the sabbatical objectives from my division/department chairs and supervisors, and securing letters of support from the host institution(s). Additional points to consider during this early stage of planning include:
  - Obtaining the recommended vaccinations and/or medications (the Centers for Disease Control and Prevention website is an excellent place to start);
  - Seeking country-specific news for the three months prior to travel;
  - Reviewing my institution’s policies related to academic leave, benefits, and health insurance while abroad; and
  - Scheduling a few presentations and meetings with potential collaborators including host country organizations working within my main areas of interest. This can be done through online communication systems such as Skype, FaceTime, or WhatsApp to facilitate face-to-face conversations. These individuals may also provide additional contacts that keep the cycle of identifying new collaborators moving forward.
3. **Build and maintain a strong research and social network.** Emailing and calling non-profit organizations, university professors with similar interests, or government agencies working in your specific field of interest may be awkward at times but it is a reasonable way to get started. Just as in most academic research settings, individuals in low-middle income countries (LMIC) are happy to talk about their work and challenges with international visitors. Perhaps the international visitors simply do not consider or ask for such opportunities enough.
4. **Take time for yourself.** While a sabbatical is still mostly work, it is designed to reinvigorate oneself. Thus, it is important to carefully consider:
  - What settles you down after a long day?
  - How do you relax?
  - How do you find peace in a new – and sometimes unsettling – environment?
  These things matter when out of the country and in need of some relaxation or piece of mind. The “I must make the most of this opportunity by working all the time” philosophy often backfires when we do not take into account the other demands on our time or the fact that experiencing another country/culture also requires time for reflection.
5. **Learn about the host country.** While we can never claim to be experts in all aspects of another country, learning as much as possible about the host country history, culture, social norms, language, and politics is essential for a successful global health sabbatical. *South African history in one hour* was a great resource for me to review key South African historical events. Additionally, I wanted to incorporate a social justice perspective by learning more about apartheid, health inequities, and race/racism in modern South Africa. A robust understanding of the history and culture helped me understand how my own culture has shaped my own personal and professional development and informs a more thoughtful and holistic approach to my research. Visits to historical and cultural sites ensured an appreciation for the context in which day-to-day life is conducted in Southern Africa. Similarly, exploring the events surrounding colonization, country building, and establishment and repeal of apartheid enhanced my understanding of health inequities that persist in today’s South Africa. Regular visits to clinical settings provided a more holistic view of the very people suffering the most from the diseases/infections I was studying. Engaging other laboratory and clinical researchers about their daily challenges helped in crafting alternative research strategies and even lead to an interesting side project or two.
6. **Consider your funding options.** Funding a sabbatical can be a significant source of stress for interested individuals. A survey of sabbatical programs in Departments of Emergency Medicine found that lack of funds or grants for research was a major obstacle for sabbaticants [1]. Nonetheless, there are a variety of funding sources to support a sabbatical, including institutional support, departmental support, grants and fellowships, teaching and research exchange programs, professional organizations, and disease-specific foundations. A short list of programs that may facilitate sabbatical experiences is provided in Appendix 1.
7. **Document the experience.** Not all of us write every day. Nonetheless, a valuable way to keep track of potential manuscript ideas is to start by taking notes at meetings and when interesting situations arise at work or during personal time. More importantly, connections between global health and other societal issues frequently require some time to emerge and coalesce. Taking notes – and keeping them all in one place – gets some of those fragmented ideas on paper while giving them time to coalesce in your mind into something bigger than just jumbled notes. Critical reflection – at a personal and professional level – was a key to my sabbatical. This helped me remember why I was doing a sabbatical rather than simply focus on what I was doing during the sabbatical.
8. **Review successes and challenges with others.** Sabbatical is most successful when others are active participants as well. Share your interesting experiences and unique challenges with interested individuals at the host institution, friends and family, as well as your home institution upon our return to the US. While the latter may occur during informal discussions, other formal opportunities include classroom lectures, Medical Grand Rounds or departmental presentations, conference presentations, invited speaker or departmental presentations at the host institution, and/or authorship on manuscripts. This is also essential for highlighting the inherent value added and providing concrete evidence of scholarly productivity associated with the sabbatical to your home institution. Many academic leaders are reluctant to encourage sabbaticals amongst their faculty for fear that they could affect the grant income or productivity. However, the inherent value of these experiences must be described and presented to leadership regularly, perhaps with attention given to faculty wellness and prevention of burnout.
9. **Challenge your assumptions and definition of the “typical way of doing things.”** As faculty, we often find ourselves reminding learners that there are multiple ways to solve a problem. Another way of saying this is that “it is not good; it is not bad; it’s just different.” However, we frequently forget this important lesson as faculty members. Identifying our assumptions about another culture (or health care system) in advance, then challenging those very assumptions regularly is an important part of any intercultural experience. In many ways, one of the most rewarding aspects of this sabbatical was having the people of South Africa and Botswana identify and describe their biggest challenges rather than attempting to do that myself. This challenged my assumptions and the cultural lens through which I see and experience this part of the world.

Several of these recommendations align well with those offered by others, including defining realistic objectives, ensuring adequate time for preparation, maintaining a flexible schedule while on sabbatical to explore new opportunities, and sharing your experience with others upon your return to your home institution [15,16]. Other distinctive aspects of a global health sabbatical include the evolving nature of global health as an academic discipline; the wide variety of clinical, service, educational, and research options to choose from; and the significant logistical considerations. While these tips may not be germane to all global health sabbaticals, they can provide an important framework for those wishing to experience this important career and personal development option.

Sabbaticals can be rich and rewarding experiences for faculty and their home institutions regardless of discipline. However, in global health, sabbatical periods may be particularly useful in allowing faculty to engage in the very work that they are teaching to their students. Faculty growth and development are essential as the growth and development of the students that we teach and engage.

## Additional File

The additional file for this article can be found as follows:

10.5334/aogh.724.s1 Appendix 1. A short list of programs that may facilitate sabbatical experiences is provided in Appendix 1.
